# Methylene Blue Bridges the Inhibition and Produces Unusual Respiratory Changes in Complex III-Inhibited Mitochondria. Studies on Rats, Mice and Guinea Pigs

**DOI:** 10.3390/antiox10020305

**Published:** 2021-02-16

**Authors:** Gergely Sváb, Márton Kokas, Ildikó Sipos, Attila Ambrus, László Tretter

**Affiliations:** 1 Laboratory of Neurobiochemistry, Department of Biochemistry, Institute of Biochemistry and Molecular Biology MTA-SE, Semmelweis University, POB. 262 Budapest, H-1444 Budapest, Hungary; svab.gergely@med.semmelweis-univ.hu (G.S.); kokas.martonn@gmail.com (M.K.); ambrus.attila@med.semmelweis-univ.hu (A.A.); 2 Department of Neurology, Semmelweis University, POB. 262 Budapest, H-1444 Budapest, Hungary; sipos.ildiko@med.semmelweis-univ.hu

**Keywords:** oxidoreduction, NAD(P)H, cytochrome c, methylene blue, bioenergetics, compartments, rodent mitochondria

## Abstract

Methylene blue (MB) is used in human therapy in various pathological conditions. Its effects in neurodegenerative disease models are promising. MB acts on multiple cellular targets and mechanisms, but many of its potential beneficial effects are ascribed to be mitochondrial. According to the “alternative electron transport” hypothesis, MB is capable of donating electrons to cytochrome c bypassing complex I and III. As a consequence of this, the deleterious effects of the inhibitors of complex I and III can be ameliorated by MB. Recently, the beneficial effects of MB exerted on complex III-inhibited mitochondria were debated. In the present contribution, several pieces of evidence are provided towards that MB is able to reduce cytochrome c and improve bioenergetic parameters, like respiration and membrane potential, in mitochondria treated with complex III inhibitors, either antimycin or myxothiazol. These conclusions were drawn from measurements for mitochondrial oxygen consumption, membrane potential, NAD(P)H steady state, MB uptake and MB-cytochrome c oxidoreduction. In the presence of MB and complex III inhibitors, unusual respiratory reactions, like decreased oxygen consumption as a response to ADP addition as well as stimulation of respiration upon administration of inhibitors of ATP synthase or ANT, were observed. Qualitatively identical results were obtained in three rodent species. The actual metabolic status of mitochondria is well reflected in the distribution of MB amongst various compartments of this organelle.

## 1. Introduction

The synthetic drug methylene blue (MB) has been used in human therapy since the last decade of the nineteenth century [1]. Its indications are extended for a broad spectrum of diseases from the treatment of toxic conditions to even neurological diseases [2]. MB is useful in one way or another, e.g., to kill cells in the course of photodynamic therapy, or help cells survive. Currently, MB is used in medicine as a photosensitizer for antibacterial disinfection [3] and MB derivatives are promising as possible anticancer agents [4]. On the other hand, MB is an established antidote in cyanide poisoning [5] and approved for the treatment of methemoglobinemia [6] MB had promising effects on animal or in vitro models of neurodegeneration [7,8]. For recent reviews, see [9,10].

Complex III is at the crossroad of the respiratory complexes, accepting electrons from α-glycerophosphate dehydrogenase, fatty acid oxidation, complex I and complex II [11]. The electron transfer mechanism inside the complex has been a subject of dispute for a very long time. The complex consists of 11 protein components, 10 are encoded by the nuclear, while 1 by the mitochondrial DNA. Two sets of the 11 protein units [12] form the functional dimer complex [13]. Until 2015 ten mutations in different proteins were associated with CIII deficiency [14]. True disease conditions are rarely associated with complex III, although the diagnosis is not easy in the absence of specific symptoms. A recent summary of these diseases conditions is reviewed by [14]. Complex III is one of the major reactive oxygen species (ROS) forming sites in the mitochondria [15,16,17]. Inhibition of CIII by antimycin or myxothiazol results in a very high rate of ROS production [18,19]. These inhibitors act on different sites of CIII’s “Q cycle” [20]. 

The mitochondrion is a target for MB: the mitochondrial connection has been explored in 255 recent scientific articles. The beneficial effects of MB exerted on respiratory chain-inhibited mitochondria are summarized in the “alternative electron transport” theory [21,22]. According to that, MB can accept electrons from NADH+H^+^ and/or from the FMN functional group of complex I and can reduce cytochrome c bypassing CI and CIII. 

Earlier, it was published by our group that MB can beneficially affect the bioenergetic parameters of mitochondria after inhibiting complex I or complex III [23]. More recently, partially contradictory results were also published by others [24]. According to their results, the inhibition of complex III by antimycin cannot be bypassed by methylene blue, because MB donates electrons proximal to the antimycin binding site of CIII in mice. The authors of the latter study argued that the contradiction between their results and ours is probably due to differences in the examined species. Surveying the literature, we were unable to find data that display crucial qualitative differences in the basic bioenergetics of mitochondria isolated from the brain of various rodent species. However, the negative results always refer to a particular problem and the generalization can be misleading.

The aim of this study was to reinvestigate whether inhibition of the mitochondrial respiratory complex III (at various sites) could perhaps be overcome or bypassed by MB. In order to assess potential species-specific characteristics, experiments were performed on three different rodent species (guinea pig, mouse and rat). It has also been investigated in model systems and in mitochondria as well, whether MB could donate electrons directly to cytochrome c. Besides corroborating our earlier results, i.e., proving the beneficial effects of MB on isolated brain mitochondria treated with CIII inhibitors; antimycin A or myxothiazol, unusual bioenergetic reactions to ADP and the adenine nucleotide translocase (ANT) inhibitor carboxy-atractylate (CAT) are described and analyzed in the presence of complex III inhibitors and MB. It is concluded that (i) MB can partially overcome the inhibition of CIII mediated by either antimycin or myxothiazol, (ii) MB can directly transfer electrons to cytochrome c, (iii) the unusual bioenergetic reactions in the presence of MB and CIII inhibitors namely an ADP-mediated inhibition of respiration, increase in NADH/NAD^+^ steady state, and the CAT-induced increase in oxygen consumption rate (OCR) and decrease in the NADH/NAD^+^ ratio are also species-independent and can potentially all be attributed to the oxidation-reduction dependent shuttling of MB between the mitochondria and the extramitochondrial space.

## 2. Materials and Methods 

### 2.1. Isolation of Brain Mitochondria

Mitochondria of synaptic and nonsynaptic origin were isolated from three different rodent species (mouse, rat and guinea pig) brain. Animals were decapitated via a procedure that is in accord with the International Guiding Principles for Biomedical Research Involving Animals and Guidelines for Animal Experiments at Semmelweis University. According to the EU Directive “ Directive 2010/63/eu of the European Parliament and of the Council of 22 September 2010 on the protection of animals used for scientific purposes” (Chapter 1, Article 3, Definitions 1, Paragraph 2: “killing the animals solely for the use of their organs or tissues”), the applied procedure above does not require any further specific permission. A discontinuous Percoll gradient was used for isolation of mitochondria, as was detailed earlier [25,26]. At the end of the centrifugation steps, the pellet was resuspended in buffer B (in mM: 225 mannitol, 75 sucrose, 5 Hepes, pH 7.4 (KOH)) to about 30 mg/mL mitochondrial protein concentration. Mitochondrial protein concentration was determined by the modified Biuret reaction [27]. Mitochondria were prepared and were used within 4 hours in each experiment. Unless otherwise indicated 0.1 mg/mL mitochondrial protein concentration was applied for most experiments.

### 2.2. Chemicals and Standard Assay Medium

All laboratory chemicals, except for ADP, were purchased from Sigma (St. Louis, MO, USA). ADP was purchased from Merck (Darmstadt, Germany). Experiments were carried out in a standard assay medium containing (in mM): 125 KCl, 20 Hepes, 2 K_2_HPO_4_, 1 MgCl_2_, 0.1 EGTA, pH 7.0 (KOH)), supplemented with 0.025% fatty-acid-free bovine serum albumin (BSA).

### 2.3. Measurement of Mitochondrial Oxygen Consumption

Mitochondrial oxygen consumption assays were performed using the high-resolution respirometry system Oxygraph-2K (Oroboros Instruments, Innsbruck, Austria) [28] at 37 °C in 2 mL chambers. Oxygen consumption rate was calculated as the negative time derivative of the oxygen concentration. Oxygen sensors were calibrated at air saturation and in oxygen-depleted medium. Mitochondria (0.1 mg/mL mitochondrial protein) were energized with pyruvate *plus* malate (5 mM each) or glutamate *plus* malate (5 mM each).

### 2.4. Measurement of Mitochondrial Membrane Potential (Δψ_m_)

ΔΨ_m_ was determined with two different methods. The first uses the cationic dye safranin O, which is accumulated and quenched in energized mitochondria [29]. The dye concentration was 2 µM. The excitation and emission wavelengths were 495 and 585 nm, respectively, as described previously [30]. Fluorescence was detected in a PTI Deltascan fluorescence spectrophotometer (Photon Technology International, Lawrenceville, NJ, USA).

In certain experiments mitochondrial membrane potential (Δψ_m_) was detected simultaneously with O_2_ consumption by Oroboros O2k equipped with the O2k-LED2 Fluo-Module, excitation LED 465 nm short-pass filter and emission long pass filter using fluorescent dye safranin O (2 µM). This approach made it possible to have entirely identical conditions for the OCR and Δψ_m_ measurements. Measurements using the dedicated fluorescence spectrophotometer (PTI Deltascan, Lawrenceville, NJ, USA) and the Oroboros O2k-LED2 Fluo-Module gave identical results.

The second method uses tetramethylrhodamine methyl ester (TMRM) in a ratiometric mode, which was described earlier [26,31]. Fluorescence was detected in the dual excitation ratiometric mode in a PTI Deltascan fluorescence spectrophotometer (Photon Technology International, Lawrenceville, NJ, USA) using excitation wavelengths of 546 and 573 nm and emission wavelength of 590 nm. The ratio of the fluorescence emitted at 590 nm using the two excitation wavelengths was plotted.

### 2.5. NAD(P)H Fluorescence Assay

NAD(P)H autofluorescence in the matrix was monitored using a PTI Deltascan fluorescence spectrophotometer (Photon Technology International, Lawrenceville, NJ, USA), as was described earlier [30]. Mitochondria (0.1 mg/mL) were incubated at 37 °C in the standard medium (see above), while fluorescence was detected via 344 nm (excitation)/460 nm (emission) wavelengths. Alterations in the NAD(P)H level were expressed as photon counts × 10^3^.

### 2.6. Measurement of Oxidoreduction State and Localization of MB

Oxidoreduction state of MB was monitored by the absorbance changes at 660 nm. MB in its oxidized form displays an absorption maximum at 660 nm. Mitochondria (0.1 mg/mL) were incubated at 37 °C in the standard medium (see above), while absorption of MB was detected at 660 nm. In order to take into account the MB-independent absorbance changes at 660 nm, measurements were repeated in the absence of MB as well, and the corresponding traces were subtracted from the traces obtained in the presence of MB.

In order to follow the localization (extramitochondrial or intramitochondrial) of MB, mitochondria (0.4 mg/mL) were incubated in the reaction medium at 1 min MB (2 µM) was added followed by pyruvate *plus* malate (5-5 mM), then by uncoupler carbonyl cyanide-*4*-(trifluoromethoxy)phenylhydrazone (FCCP) (250 nM). Absorbance (660 nm) was detected (incubation with mitochondria). After the addition of MB, pyruvate *plus* malate and FCCP aliquots were taken from the incubations (aliquots 1, 2, and 3) centrifuged for 2 min at 14,300× *g* and absorbance at 660 nm was measured (supernatant). Cuvettes containing the supernatants were bubbled with oxygen to fully oxidize MB (oxygen treatment), then treated with dithionite to fully reduce MB (dithionite). Absorbance at 660 nm was detected by a JASCO V-650 spectrophotometer (ABL&E-JASCO, Tokyo, Japan).

### 2.7. Statistical Analysis

Data are presented as the means ± S.E.M. Statistical differences were evaluated with ANOVA (SIGMASTAT; Systat Software Inc., San Jose, CA, USA) followed by the Bonferroni’s post-hoc test for multiple comparisons, or for data not following normal distribution with ANOVA on ranks Kruskal–Wallis test was applied. Student’s t-tests were used for simple comparisons. Values of *p* < 0.05 were considered to be significant.

## 3. Results

Below, results that were obtained utilizing three different rodent species are presented. Where it could be justified, however, data from only a single species are reported (to limit the length of the article). In these cases, the results obtained using the other species are available in the Supplementary Materials. The purpose of this apparently random presentation is to illustrate that measurements performed on different rodent species can be fitted into the same puzzle without changing the general conclusions of the paper.

### 3.1. Effects of Methylene Blue (MB) on the Oxygen Consumption of Complex III-Inhibited (Antimycin or Myxothiazol) Mitochondria Supported by Two Substrates in Three Different Species

Oxygen consumption is a sensitive parameter of mitochondrial bioenergetics. Oxygen consumption was measured in three rodent species (rat, mouse and guinea pig) applying either of two complex III inhibitors (antimycin or myxothiazol) and two respiratory substrates (pyruvate *plus* malate (PM) or glutamate *plus* malate (GM)) in combination and with ADP, the adenine nucleotide translocase (ANT) inhibitor carboxyatractylate (CAT) or ATP synthase inhibitor oligomycin (oligo) as well as the uncoupler FCCP.

**Figure 1:**
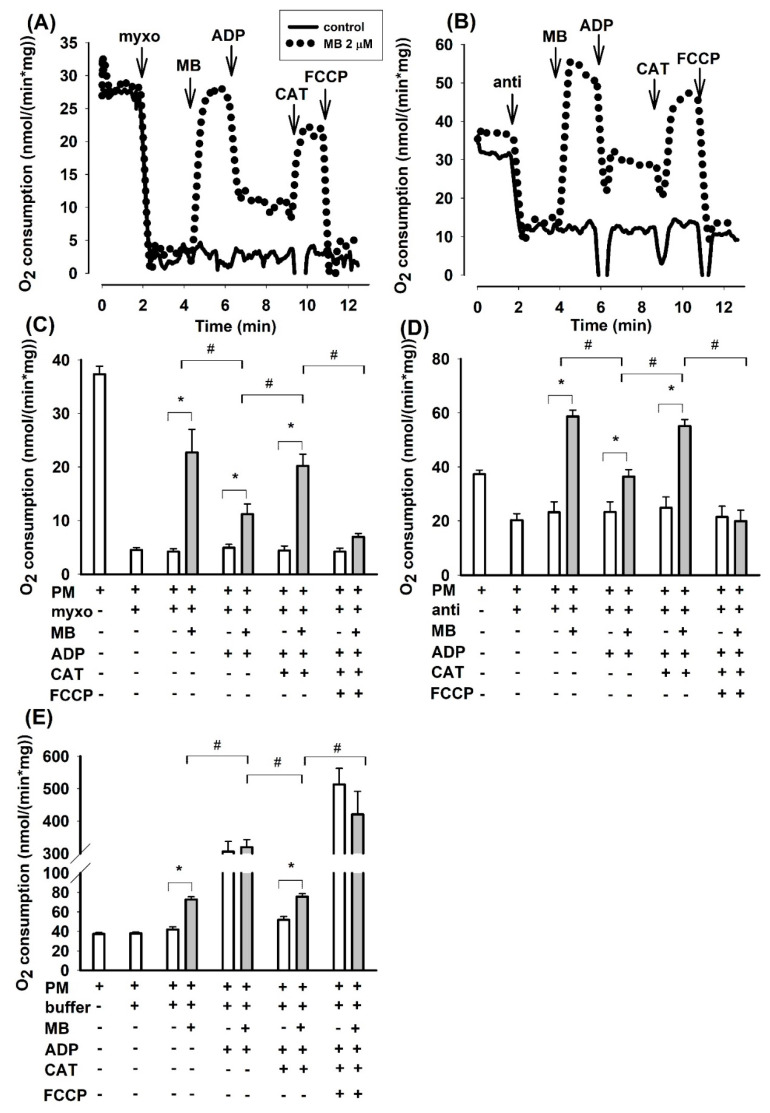
Effects of methylene blue (MB) (2 µM) on the rate of oxygen consumption in complex III-inhibited **mouse** mitochondria. In pyruvate *plus* malate (5-5 mM) supported mitochondria myxothiazol (1 µM) (**A**,**C**) or antimycin A (0.3 µM) (**B**,**D**) was applied for inhibiting of complex III. In (**E**), in the absence of inhibitors, buffer or MB was added. Representative original traces are shown in panels A and B. For traces denoted with black line inhibitor was only added, for traces represented with dotted lines, inhibitor and MB were both added. Further additions were as indicated. In (**C**,**D**,**E**), bars represent the average rates of oxygen consumption in nmol/min/mg protein ± S.E.M. (*n* = 8). * and # designate significant difference (*p* < 0.05) relative to the conditions indicated.

In agreement with the conclusions drawn from our earlier report on guinea pig mitochondria [23], the oxygen consumption rate (OCR) was inhibited again by antimycin in mouse brain mitochondria supported by PM. Addition of MB (2 µM) to antimycin-treated mitochondria stimulated the oxygen consumption by 60% (from 36.7 ± 2.0 to 58.7 ± 2.3 nmol/min/mg protein Figure 1D). The addition of ADP, however, did not significantly alter the OCR of antimycin treated mitochondria, whereas it unexpectedly decreased the rate of oxidation (from 58.7 ± 2.3 to 36.4 ± 2.6 nmol/min/mg protein) in mitochondria treated with antimycin and MB. Inhibition of the ANT by CAT significantly stimulated the rate of respiration (by 51% from 36.4 ± 2.6 to 55.1 ± 2.4 nmol/min/mg protein) in mitochondria treated with antimycin-MB-ADP. Stimulation of respiration by the ANT inhibitor was an unusual phenomenon. The addition of the uncoupler FCCP had an inhibitory effect on oxygen consumption. In the absence of MB, the antimycin-inhibited respiration was unaffected by either ADP or CAT.

Considering that antimycin in the applied concentration did not completely inhibit the mitochondrial oxidation, the effects of another well-known inhibitor of complex III were also studied. The sites of inhibition within complex III are different for the two inhibitors [20]. Myxothiazol exerted a more pronounced effect on the PM-supported oxygen consumption; the degree of inhibition was 88% (from 36.7 ± 2.0 to 4.5 ± 0.6 nmol/min/mg Figure 1C). MB (2 µM), however, increased the rate of oxidation by a factor of five (from 4.5 ± 0.6 to 22.7 ± 4.3 nmol/min/mg). Similar to that observed in antimycin plus MB treated mitochondria, added ADP decelerated the oxygen consumption whereas CAT stimulated it (Figure 1C). Representative traces are shown in Figure 1A,B.

Consistent results could be obtained from experiments, conducted under identical conditions, addressing the oxygen consumptions of guinea pig or rat brain mitochondria. Bar charts of the summarized results are shown for guinea pig (Figure S1) and rat (Figure S2) mitochondria in the Supplementary Material. Exchanging the pyruvate *plus* malate substrate combination to glutamate *plus* malate support, the latter did not affect the fundamental findings: inhibition of complex III by either myxothiazol or antimycin hampered the oxidation of complex I substrates, while MB could relieve this effect; in most cases, with MB the rate of oxidation was higher than the basal respiration detected in the absence of complex III inhibitors (myxothiazol or antimycin) in substrate-supported mitochondria.

The unusual pattern of oxidation detected in the presence of MB and either myxothiazol or antimycin made us initiate studies for detecting potential changes in Δψ_m_ in order to clarify the mechanism behind the ADP-induced decrease and CAT-evoked stimulation of oxygen consumption.

### 3.2. Membrane Potential (ΔΨm) in Complex III-Inhibited (Myxothiazol or Antimycin) Mitochondria Is Partially Restored by MB in Mice and Rats

Δψ_m_ was semiquantitatively measured using two fluorescent methods. Using safranin fluorescence is simple and the measurement provides; reliable results. Safranin fluorescence is decreased with the hyperpolarization of Δψ_m_. TMRM fluorescence was also measured in order to avoid various possible optical artifacts. The dual excitation ratiometric mode of the TMRM fluorescence measurements gives mirror images using the two excitation wavelengths [31]. TMRM fluorescence ratio is increased with the hyperpolarization of Δψ_m_.

#### 3.2.1. Measurement of Δψ_m_ Using the Fluorescent Dye Safranin O

**Figure 2:**
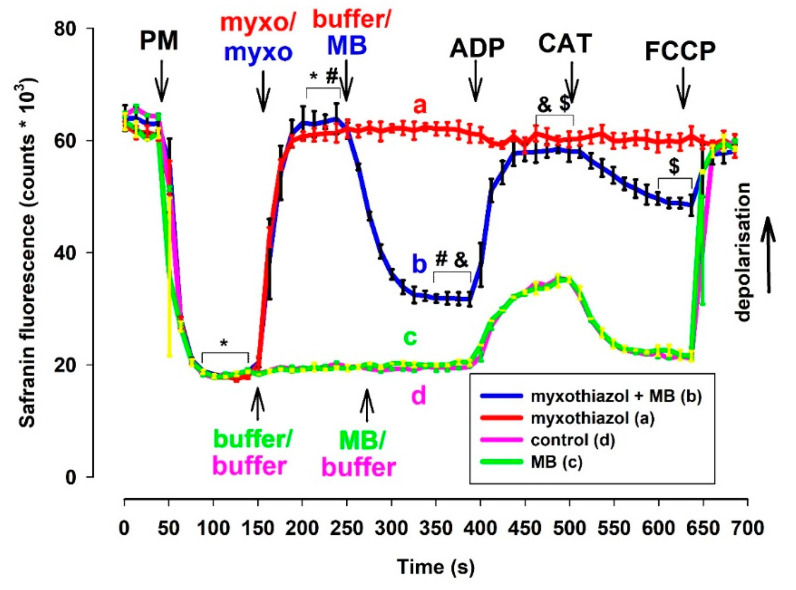
Effects of MB (2 µM) on the membrane potential in complex III-inhibited **mouse** mitochondria. Δψ_m_ was measured via safranin O fluorescence as described in Materials and Methods. Safranin was applied to pyruvate *plus* malate (5-5 mM) supported mitochondria; myxothiazol (1 µM) was used for inhibiting complex III. Trace a, red: inhibitor (myxothiazol) was added, trace b, blue: inhibitor and MB were added, trace c, green: MB was added, and trace d, pink: control condition. Further additions were: ADP (2 mM), CAT (2 µM), FCCP (250 nM), all as indicated. Traces represent the average safranin signals ± S.E.M at *n* = 3. *, #, &, $ represent significant difference (*p* < 0.05) relative to the conditions indicated.

The experimental protocol for the determination of Δψ_m_ by safranin was identical to that of the oxygen consumption measurements. In fact, in many cases, these two parameters were measured using the same cuvette equipped with an oxygen electrode and fluorescence light source/sensor unit. Complex III inhibitors almost completely depolarized the mitochondria that were supported by NADH-generating substrates. ADP did not alter the low Δψ_m_ in electron transport chain (ETC) impaired mitochondria. The ANT inhibitor CAT only induced moderate repolarization, which was afterward abolished again by the uncoupler FCCP. In complex III-inhibited mitochondria, MB that was added after the inhibitors induced significant repolarization (Figure 2). The addition of ADP induced depolarization (like in non-inhibited mitochondria), and this depolarization was followed by a polarization event evoked by the ANT inhibitor CAT. Eventually, the uncoupler FCCP completely dissipated Δψ_m_. The difference in fluorescence induced by MB proved to be statistically significant in inhibitor-treated mitochondria. Similarly, the ADP-induced depolarization and CAT-evoked polarizations were also statistically significant. Experiments utilizing rats (Figure S3) and either of the two inhibitors provided similar results. To verify the results obtained with safranin O and exclude potential optical artifacts, experiments were also performed utilizing another fluorescent dye (TMRM).

#### 3.2.2. Determination of Δψ_m_ via TMRM Fluorescence

**Figure 3:**
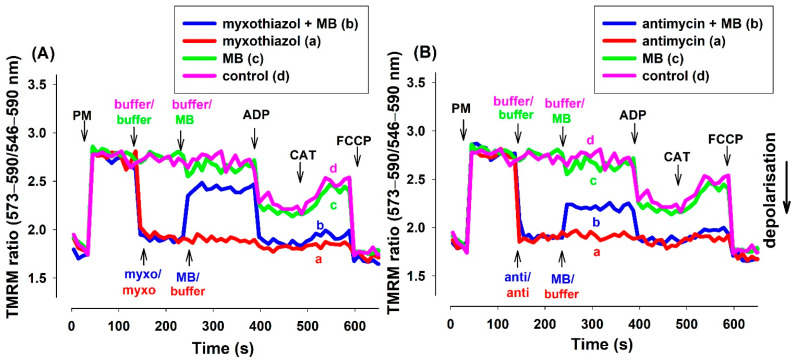
Effects of MB on the membrane potential (Δψ_m_) of the complex III-inhibited (**A**): myxothiazol, (**B**): antimycin treated **guinea pig** mitochondria. Δψ_m_ was measured by tetramethylrhodamine methyl ester (TMRM) fluorescence as was described in Materials and Methods. The standard medium (see Materials and Methods) with TMRM (100 nM) was applied to the pyruvate *plus* malate (5-5 mM) supported mitochondria. Trace a, red: myxothiazol (myxo, 1 µM) or antimycin (anti, 0.3 µM) was added, trace b, blue: inhibitors and MB (2 µM) were added, trace c, green: MB was added, trace d, pink: buffer was only added. Further additions were as follows: ADP (2 mM), CAT (2 µM), and FCCP (250 nM), all as indicated. Traces are representatives of three independent measurements.

TMRM (in ratiometric mode) and safranin provided essentially identical results in fluorescence measurements (Figure 3). Experiments utilizing rats (Figure S4) or mice (Figure S5) and either of the two inhibitors provided similar results.

### 3.3. Measurement of the NAD(P)H Steady State

**Figure 4:**
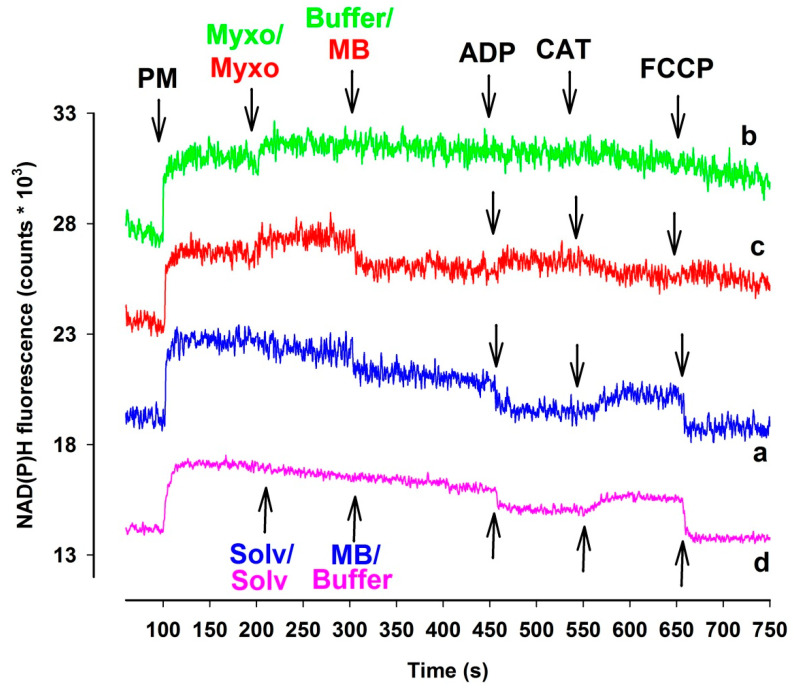
The effects of MB (2 µM) on NAD(P)H steady state of complex III-inhibited **rat** mitochondria. In pyruvate *plus* malate-supported ((PM) 5-5 mM) mitochondria myxothiazol ((myxo) 1 µM) was employed for inhibiting complex III. Trace a, blue: MB was added; trace b, green: inhibitor (myxo) was added; trace c, red: myxo and MB were added, trace d, pink: solvent and buffer were added. Further additions were as indicated. Traces are representatives of at least three independent experiments and are offset for clarity.

Alterations in the NAD(P)H/NAD(P)^+^ steady state can provide a reliable assessment of the redox homeostasis of mitochondria. Inhibition of Complex I, III, and IV may affect the NAD(P)H homeostasis. Proximal to the block the oxido-reduction steady states of the reducing equivalents NADH/NAD^+^ and FADH_2_/FAD are expected to be shifted towards the respective more reduced forms [32]. The above-mentioned reducing equivalents are predominant electron donors for MB [21,23]. If MB were able to bridge the complex III inhibitors mediated reduction of the electron transport chain, this should also be reflected in somewhat attenuated NAD(P)H signals. Therefore, in complex III inhibited mitochondria, a certain degree of oxidation of NAD(P)H was indeed expected. Antimycin has its own optical signal at the wavelengths used in these experiments, therefore only the results obtained with myxothiazol-inhibited mitochondria are presented here (Figure 4). In the presence of respiratory substrates pyruvate *plus* malate the NAD(P)^+^ pool became promptly reduced. In the absence of myxothiazol (trace a, blue line), MB and the subsequently administered ADP both induced a marked degree of oxidation of NAD(P)H, while inhibition of the ANT by CAT reduced the pyrimidin nucleotide pool back again. Analogous results were obtained in the absence of MB (trace d, pink line). In the absence of MB, myxothiazol further increased the NAD(P)H level, however, the complex III-inhibited mitochondria did not react to ADP and CAT, the NAD(P)H level remained steady, and only FCCP induced a slow decrease in the NAD(P)H fluorescence (trace b, green line). In mitochondria treated with both myxothiazol and MB (trace c, red line), after the myxothiazol-evoked increase of NAD(P)H steady state addition of MB resulted in a drop of NAD(P)H level. The addition of ADP contrary to that shown in trace a increased the NAD(P)H fluorescence, however, CAT oxidized the part of NAD(P)H pool. It is concluded that both myxothiazol and MB has an effect on the NAD(P)H level and the two compound applied in the same experimental results unexpected effects on the NAD(P)H redox equilibrium. Experiments performed with either guinea pigs (Figure S6) or mice (Figure S7) provided similar results.

### 3.4. Oxido-Reduction of MB in the Presence or Absence of Inhibitors

**Figure 5:**
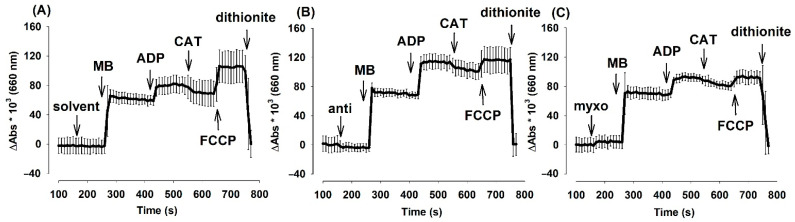
The effects of complex III-inhibitors on oxidoreduction steady state of MB (2 µM) in **mouse** mitochondria. Experiments were performed as described in Materials and Methods. In pyruvate *plus* malate-supported (5-5 mM) mitochondria absorbance differences (Abs_660_ × 10^3^) were detected in the presence or absence of MB in (**A**): uninhibited (**B**): myxothiazol (**C**): antimycin treated mitochondria. Further additions were as indicated. Curves represent the average of three independent experiments ± S.E.M.

The observed changes in oxygen consumption, membrane potential and NAD(P)H level as a consequence of administering complex III inhibitors and MB appeared to contradict one another. Usually, ADP depolarizes the Δψ_m_ and decreases the NAD(P)H level. Moreover, the inhibition of ANT generally decelerates the oxygen consumption while hyperpolarizing the mitochondria and increasing the NAD(P)H concentration. However, under the conditions applied in our experiments, we observed unexpected responses. In order to resolve these contradictions, we also attempted to determine the oxidoreduction state of MB on the basis of the distinct spectral signatures of its oxidized and reduced forms. MB in its oxidized form displays the maximum absorbance at 660 nm. Experiments for Figure 5. utilized the following protocol (similarly to Figure 1, Figure 2, Figure 3 and Figure 4.): mitochondria, substrate, inhibitor, MB, ADP, CAT, FCCP. Experiments were terminated by adding dithionite; this compound reduced and hence discolored MB.

In order to take into account the changes of absorbance at 660 nm in the absence of MB, traces obtained in the absence of MB at 660 nm were subtracted from the traces obtained in the presence of MB. Summary curves depicted in Figure 5. demonstrate that the addition of MB in either the presence or absence of complex III inhibitors resulted in a large increase in the absorbance at 660 nm, which wavelength is attributed to the oxidized form of MB. ADP evoked the oxidation of MB, inhibition of the ANT partially re-established the previous steady state, in the presence of FCCP MB became more oxidized, and sodium dithionite reduced and discolored the MB pool. Analogous results were obtained with mitochondria isolated from the guinea pig brain as well (Figure S8). Traces without subtraction of MB-free absorbance are presented in Figure S9.

### 3.5. Changes in Mitochondrial Bioenergetics May Modify the Compartmentalization of MB

**Figure 6:**
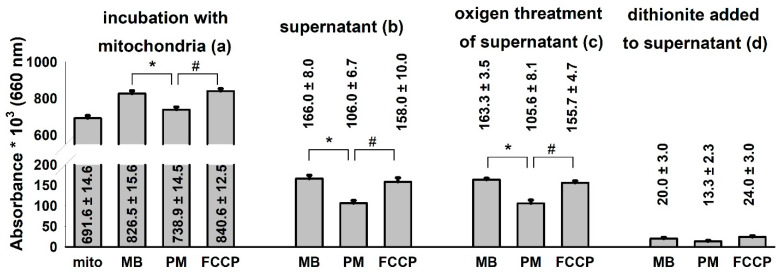
Distribution of MB among mitochondrial and non-mitochondrial compartments in various energetic conditions of the organelle. **Guinea pig** mitochondria were incubated in the standard assay medium as was described in the Materials and Methods. Additions were as follows MB (2 µM) at 1 min; PM 5-5 mM at 3 min and FCCP 250 nM at 5 min, and the absorbances at 660 nm were measured. Two minutes after each addition, aliquots were collected, mitochondria were sedimented and the absorbance of the supernatant was determined. Then, O_2_ gas was bubbled through all of the supernatant samples for 2 min, and a subsequent reduction step was also carried out by using excess sodium dithionite. Absorbances were also recorded after performing the O_2_ and dithionite treatments. Curves represent the average of ten independent experiments ± S.E.M. *, # represent significant differences (*p* < 0.05) relative to the conditions indicated.

It is very likely that MB given to isolated mitochondria is not localized entirely within the mitochondrial compartments. It is known that the oxidized MB, which possesses an overall positive charge, can be taken up by mitochondria [33]. Oxidized MB has an absorption maximum of 660 nm. Experiments were performed to describe the distribution and oxidoreduction state of MB added to non-energized, respiratory substrate-supported and uncoupled mitochondria isolated from guinea pig brain (Figure 6).

The addition of MB (2 µM) to substrate-free mitochondria leveled up the absorbance of the solution at 660 nm. Energization of mitochondria with the respiratory substrate pyruvate *plus* malate decreased the absorbance indicating the reduction of MB. The uncoupling of the mitochondria with FCCP shifted the [oxidized]/[reduced] ratio of MB towards the more oxidized state (Figure 6a). Aliquots were collected and sedimented from the mitochondrial incubations and the absorbance of the mitochondrion-free supernatants was determined (Figure 6b). The lowest absorbance was measured in the supernatants of energized mitochondria. An equally high level of MB could be detected in the supernatants of the substrate-free and uncoupled mitochondria. In order to oxidize the MB of the supernatants completely, gas oxygen was bubbled through the cuvettes. There was no significant change detected between the non-oxygenated and the oxygen-saturated samples indicating that the majority of MB in the supernatant was in the oxidized form. Reducing agent sodium dithionite was applied to the same samples and a significant decrease of 660 nm absorbance was detected, indicating that the colored substance was indeed the MB (Figure 6d). Results depicted in Figure 6. indicate that MB is taken up into energized mitochondria and will be reduced there. Deenergetization of mitochondria by uncoupler FCCP results in an almost complete release of MB decisively in the oxidized form.

The aim of the following experiments was to give support to the hypothesis that MB could stimulate the transfer of electrons from NADH to cytochrome c (cyt. c).

### 3.6. Reducing Equivalent NADH Can Reduce Cytochrome c in the Presence of MB

#### 3.6.1. Cytochrome c Reduction Is also Stimulated by MB in the Presence of Complex III Inhibitors as Well

**Figure 7:**
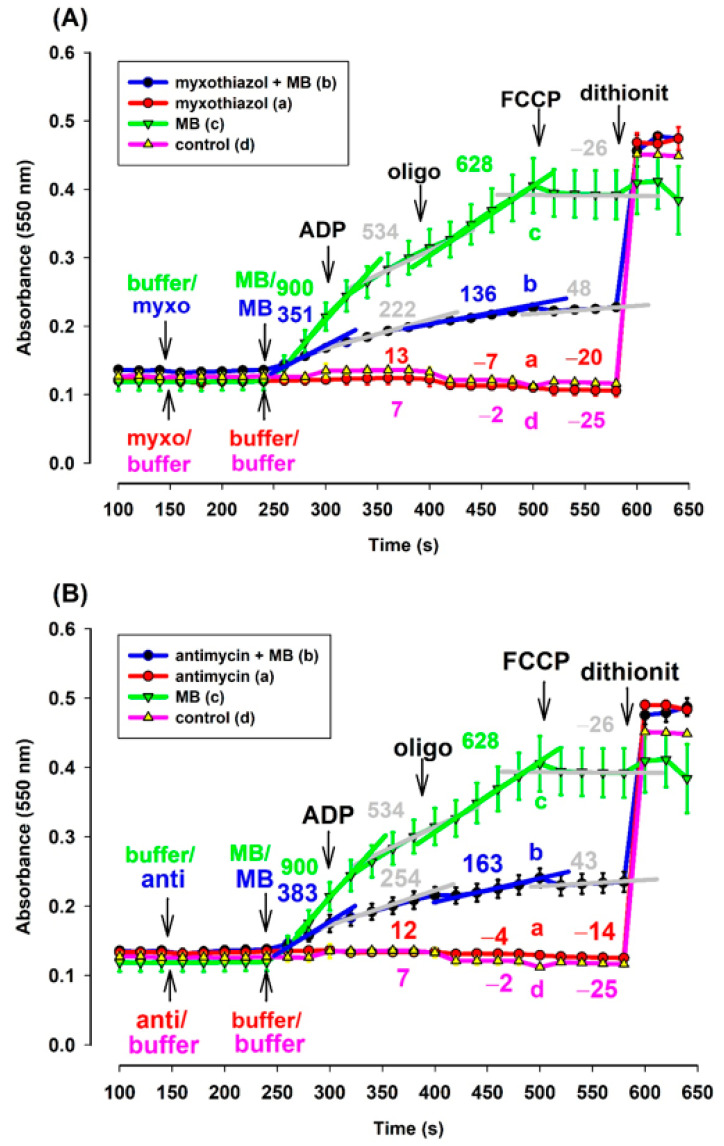
Effects of MB on the reduction of exogenous ac-cyt. c in the complex III-inhibited (**A**): myxothiazol, (**B**): antimycin treated **guinea pig** mitochondria. Reduction of exogenous ac-cyt. c was measured at 550 nm wavelength. The standard medium (see Materials and Methods) was supplemented with ac-cyt. c (25 µM). Guinea pig brain mitochondria were supported by pyruvate *plus* malate (5-5 mM). Trace a, red: myxothiazol (myxo, 1 µM) or antimycin (anti, 0.3 µM) was added, trace b, blue: inhibitors and MB (0.5 µM) were added, trace c, green: MB was added, trace d, pink: buffer was only added. Further additions were as follows: ADP (2 mM), CAT (2 µM), and FCCP (250 nM), all as indicated. Numbers above the curves indicate the rate of absorbance changes ΔA_550_/s × 10^−6^. Straight lines were fitted to various parts of the curves and slopes were calculated. Traces are averages of three independent measurements ± S.E.M.

Mitochondria isolated from guinea pig brain were supported by pyruvate *plus* malate. Exogenous acetyl-cytochrome c (ac-cyt. c) was present in the reaction medium from the beginning of the experiments. In the absence of MB and in either the presence or absence of CIII inhibitors, reduction of ac-cyt. c could not be observed (Figure 7A,B curves (a) and (d)). However, in the presence of MB, the energized mitochondria were able to reduce cyt. c, even in the presence of CIII inhibitors (Figure 7). The rate of reduction, however, was different in the presence (trace b, blue) and absence (trace a, green) of the inhibitors. In the absence of the inhibitors, the addition of MB provoked a higher rate of cyt. c reduction. ADP lowered the rate of cyt. c reduction in both the absence and presence of the inhibitors. The ATPase inhibitor oligomycin stimulated the cyt. c reduction, while depolarization of the inner mitochondrial membrane reversed that. This experiment proved that exogenous ac-cyt. c can be reduced by MB.

#### 3.6.2. In the Presence of NADH MB Is Able to Reduce Cytochrome c In Vitro

**Figure 8:**
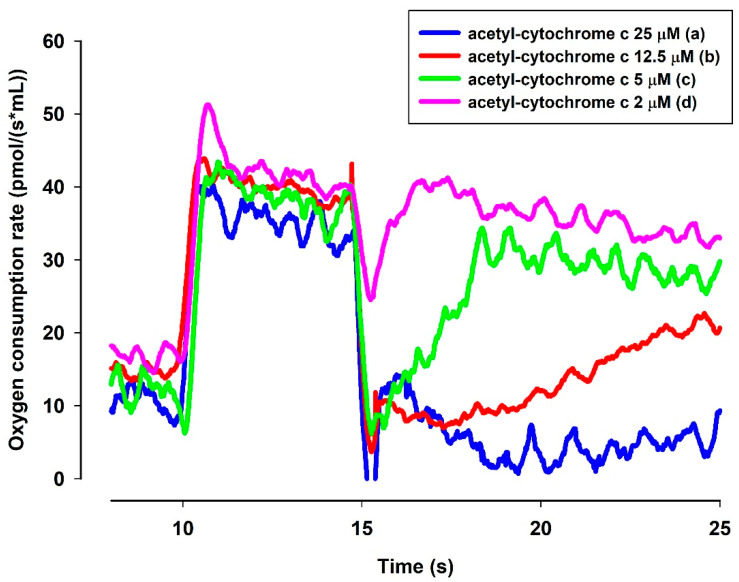
Effects of MB and ac-cyt. c on the oxygen consumption in the presence of NADH. The standard medium (see Materials and Methods) was supplemented with NADH (250 µM). All of the traces were supported by MB (2 µM) and ac-cyt. c (trace a, blue 25 µM; trace b, red 12.5 µM, trace c, green 5 µM, trace d, pink 2 µM). Traces are representatives of three independent measurements.

A question to be answered was whether the reduced MB is able to reduce cyt. c. In order to elucidate this, an NADH solution in the standard medium was examined with a high-resolution oxygen electrode to determine the actual oxygen concentration. The oxygen consumption of this solution was moderate. Upon administering MB, the oxygen consumption rate was immediately increased. The rate of oxygen consumption was gradually decreased as NADH was oxidized by MB (Figure 8). When ac-cyt. c was added, the oxygen consumption rate was immediately dropped then started to increase more slowly than in the absence of cyt. c. When reaching a plateau (attributed to the near-complete reduction of ac-cyt. c), the slopes of the two curves (traces c and d) became equal. This experiment indicated that MB was able to accept electrons from NADH and in the absence cyt. c it reacted with molecular oxygen. This is reflected in the enhanced oxygen consumption in the presence of NADH and MB. Ac-cyt. c, which is less prone to autoxidation, could react with MB, and this reaction decelerated the autoxidation of MB by O_2_. As ac-cyt c became more and more reduced, it eventually stopped accepting electrons and the two rates became equal (traces c and d). The results presented above, however, provided indirect proof for the participation of cyt. c in the oxidation of NADH.

#### 3.6.3. NADH-Dependent Reduction of Acetylated Cytochrome c in the Presence of MB

**Figure 9:**
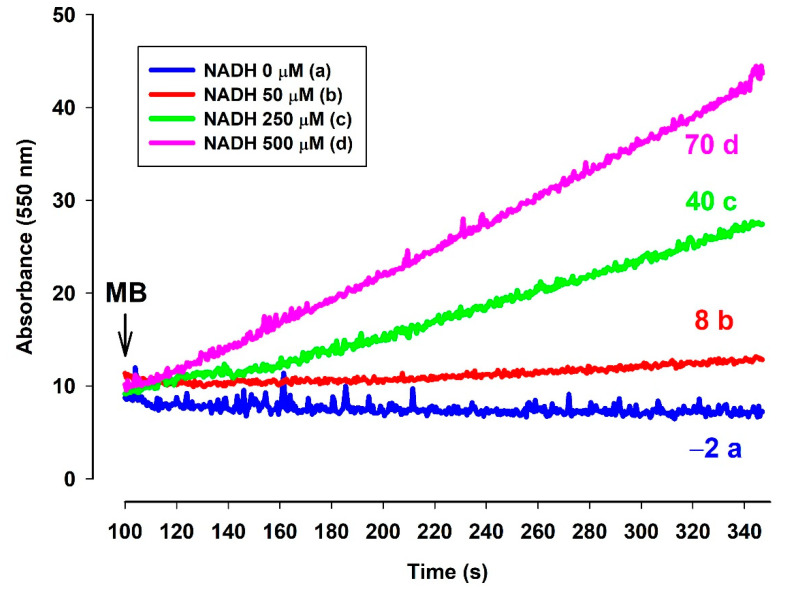
Effects of MB on the reduction of ac-cyt. c in the presence of NADH in various concentrations. Reduction of ac-cyt. c was measured at 550 nm wavelength. The standard medium (see Materials and Methods) was supplemented with ac-cyt. c (25 µM) and NADH (trace a, blue 0 µM; trace b, red 50 µM; trace c, green 250 µM; trace d, pink 500 µM). The numbers above the traces indicate the rate of ac-cyt c reduction (ΔA_550_/s × 10^−6^). Traces are representatives of three independent measurements.

In order to detect directly the electron transfer between MB and cyt. c, the reduction of cyt. c by MB was measured (Figure 9). In the absence of NADH no substantial reduction of ac-cyt. c could be observed (Figure 9. trace a, blue). However, the addition of MB (2 µM), evoked a high rate of ac-cyt. c reduction, the rate was dependent upon the NADH concentration.

## 4. Discussion

### 4.1. Can MB Relieve the Inhibition of Complex III?

MB induced a significant increase in oxygen consumption in mitochondria treated with either antimycin or myxothiazol. These results are in good agreement with our previous findings, while they are quite inconsistent with those reported recently by another group [24]. In the present study, mitochondria isolated from three different animal species were investigated and the drawn conclusions were nearly identical in all three systems. MB was able to stimulate the oxygen consumption in mitochondria treated with either antimycin or myxothiazol. In agreement with this, MB was also able to partially restore the Δψ_m_, which was depolarized earlier by either inhibitor of complex III. In order to eliminate as much as possible the potential optical artifacts, two fluorescent dyes were utilized for detecting alterations in Δψ_m_. The two applied dyes both produced concordant results in all the three investigated species (safranin: Figure 2. mouse; Figure S3. rat, TMRM: Figure 3. guinea pig, Figure S4. rat, Figure S5. mouse). The validity of the results was further corroborated by the subsequent additions of ADP and the ANT inhibitor CAT. The addition of ADP to the inhibitor-treated but MB-rescued mitochondria depolarized the mitochondrial membrane, while the subsequent addition of CAT triggered polarization. The partial rescue of oxygen consumption as well as Δψ_m_ indicated that MB could indeed be beneficial under those pathological conditions that affect complex III.

### 4.2. Which Particular Segment of the Respiratory Chain Could Oxidize MB?

Results depicted in Figure 1. (and Figures S1 and S2) indicated that MB was able to restore the respiratory activities that were previously impaired by CIII inhibitors. However, during this process, mitochondria were unable to completely reclaim the Δψ_m_ that could be detected in the absence of an inhibitor. These results can potentially be accounted for by several mechanisms: (i) since MB is also a well-known photosensitizer and hence ROS producer [3,24,34], it is possible that ROS formation by MB could be responsible for the MB-induced oxygen consumption. However, the H_2_O_2_ formation detected in the presence of MB was more than one order of magnitude slower than the augmentation in the respective oxygen consumption (Figure 1.) calculated from [23]. The MB-induced bypassing of the inhibited complex III would lower the efficacy of the electron transfer system, from the 10H^+^/2e^−^ to 2H^+^/2e^−^. This attenuation in the efficacy of proton gradient formation could permit a higher rate of electron transport with less Δψ_m_ built up. Indeed, in mitochondria inhibited by antimycin, MB could induce faster oxygen consumption than the inhibitor-free state. Although the membrane potential was not entirely re-established, a significant increase could still be detected. This and the simultaneously enhanced oxygen consumption indicated that the highly reduced electron transfer system behind the block in the presence of MB could transfer electrons to electron acceptors shunting the block and the restarted electron transfer was associated with a higher rate of proton extrusion, i.e., with the partial repolarization of Δψ_m_. 

According to Gureev et al. [24], the reduced form of MB could potentially donate electrons to complex III at the Q_o_ site and that would be the reason why inhibition of CIII by antimycin (acting at the internal Q_i_ site) could not be bypassed by MB. However, in the present study, when applying either the Q_o_ site inhibitor myxothiazol or the Q_i_ inhibitor antimycin, MB proved to be equally effective. Nevertheless, our data do not exclude the possibility that in the absence of CIII inhibitors MB could potentially still donate electrons to the Q_o_ site. However, perhaps it is an even more important question whether MB is capable of donating electrons downstream to complex III?

### 4.3. Reduced MB Transfers Electrons to Cytochrome c

#### 4.3.1. MB Is Able to Reduce Exogenous ac-cyt. c in the Presence of Mitochondria

In order to assess the potential oxidation of reduced MB by cyt. c, exogenous ac-cyt. c was added to isolated mitochondria and the same protocol used in previous experiments was applied. Mitochondria were supported by respiratory substrates, CIII inhibitors and subsequently, MB was administered, which was followed in order by ADP, oligomycin and FCCP. With no MB present, no cyt. c reduction could be detected. However, MB stimulated ac-cyt. c reduction regardless of the presence of CIII inhibitors. This experiment unambiguously confirmed that even exogenous ac-cyt. c can be reduced by MB. Considering that the cyt. c concentration inside the mitochondrion [35] is at least ten times higher than the one applied in the above experiments (25 µM), it is very likely that endogenous cyt. c would also be capable of substantially oxidizing the reduced MB. 

The essential criterion for this is the free transfer of MB across the mitochondrial membranes. According to our hypothesis, the uptake of oxidized MB is stimulated by the inside negative Δψ_m_. The positive charge of the oxidized MB will be neutralized by the reduction. The reduced MB will not be retained by the membrane potential and it will not be repulsed by the positive charges of the lysine residues of cyt. c anymore, either, therefore MB could indeed reduce cyt. c. Upon oxidation, MB regains its positive charge, thus dissociates from cyt. c and participates in a new redox cycle. This assumption is very much supported by the results of Gabrielli et al. [33].

#### 4.3.2. In Vitro Reduction of Cytochrome c in the Presence of NADH and MB

In the presence of NADH, the slow autooxidation of NADH resulted in a certain degree of steady oxygen consumption (see Figure 8). The addition of MB enhanced the oxygen consumption and hence the autooxidation of NADH. Ac-cyt. c in various concentrations decelerated the oxygen consumption implying that reduced MB can indeed transfer electrons to cyt c and the low rate of autoxidation of ac-cyt c can (transiently) lower the rate of superoxide formation. In the absence of other electron acceptors, O_2_ can be reduced to superoxide, while the spontaneous dismutation of the latter will lead to the formation of H_2_O_2_. This experiment is an indirect proof for the electron transport from NADH to MB and from reduced MB to cyt c. Reduction of MB and ac-cyt c by NADH was also demonstrated in Figure 9. In the presence of MB (2 µM), the rate of ac-cyt c reduction was directly proportional to the NADH concentration. Similar results have already been published before [36], however, in the present context, it was necessary to confirm whether reduced MB is able to reduce ac-cyt c. These experiments again directly strengthened the notion that MB might indeed be beneficial in mitigating the bioenergetic burden of complex III inhibition. Even though the residual oxygen consumption may not afford to generate a proton gradient, the increased rate of respiration, which is a consequence of the increased rate of oxidation of reducing equivalents, would result in an increased rate of citric acid cycle. The latter, even with inactive oxidative phosphorylation machinery present, could produce a certain amount of ATP via substrate-level phosphorylation [37].

### 4.4. Unusual Oxidation Pattern in CIII Inhibitor and MB Treated Mitochondria

Finally, a potentially important aspect of this study was to describe and understand the unusual changes in respiration and NAD(P)H steady state detected in the presence of respiratory chain complex III inhibitors and MB.

Usually, the addition of ADP to mitochondria supported by respiratory substrates initiates an increased rate of respiration as a consequence of the OXPHOS-induced decrease in the membrane potential, lower NADH/NAD^+^ ratio and stimulation of the citric acid cycle. In agreement with this, inhibition of the adenine nucleotide transport, more specifically the ATP/ADP exchange carrier, or the inhibition of ATP synthase by oligomycin would have the opposite effects and the ADP-stimulated respiration would decelerate, Δψ_m_ would be hyperpolarized, and the NADH/NAD^+^ ratio would increase. We detected all of these changes under control conditions, however, in mitochondria treated with CIII inhibitors and MB, the addition of ADP although decreased Δψ_m_, as was expected, but, in parallel, the oxygen consumption was lowered (Figure 1) and the NAD(P)H level was increased (Figure 4). The addition of the ANT inhibitor CAT hyperpolarized the mitochondria, but also stimulated the oxygen consumption and decreased the NADH level. These particular results can be explained by the changes in the oxidoreduction state of MB and the shuttle/distribution of MB between the mitochondria and the surrounding environment. The data in Figure 6. indicate the changes in the localization and oxidoreduction state of MB under the conditions of a simple mitochondrial experiment. When MB was added to non-energized mitochondria, it was not taken up to an experimentally measurable degree. The addition of the respiratory substrates initiated the uptake of MB. Dissipating Δψ_m_ by an uncoupler decreased the attachment of MB to mitochondria. An approximate calculation using the molar extinction coefficient of MB (71.547 mM^−1^ cm^−1^) was made to estimate the uptake of MB in energized mitochondria. The addition of 2 µM MB to non-energized mitochondria (0.4 mg/mL) caused an increase of 0.135 in A_660_, corresponding to 1.8 µM MB; the calculated and the given [MB] were in quite good agreement (~10% difference). Energization of mitochondria by PM decreased the concentration of oxidized MB both in the presence of mitochondria and in the supernatant; absorbance of the supernatant decreased by 34%. Calculating the uptake of MB from these results, using 1 µL/mg protein for mitochondrial volume (as a rough estimation), we could conclude that in the hyperpolarized mitochondria the MB concentration was more than two orders of magnitude higher than in the extramitochondrial space. The untaken MB remained oxidized or became oxidized during centrifugation, since saturating the mitochondrial supernatant solution with oxygen did not increase the absorbance at 660 nm. Based on the conclusion that mitochondria can take up MB in its oxidized form and partially release it in its reduced form, we could attempt to interpret the unconventional results of our experiments. By detecting the absorbance changes of MB in the presence of CIII inhibitors, we could monitor the oxidoreduction state of MB.

To summarize our explanation: In the absence of inhibitors and MB, ADP attenuated the membrane potential, increased the oxygen consumption and decreased the NAD(P)H fluorescence, whereas CAT hyperpolarized the membrane, decreased the oxygen consumption and increased the NAD(P)H level.

In the absence of inhibitors, but in the presence of MB, MB in itself (in the dosage applied here at least) did not alter the membrane potential [23], but stimulated the resting (ADP-free) oxidation and decreased the NAD(P)H fluorescence diverting electrons from NADH to cyt c. For these electrons, the stoichiometry of the number of protons pumped out per pair of electrons will decrease from 10H^+^/2e^−^ to 2H^+^/2e^−^. 

With CIII inhibitors, but no MB present, ADP and CAT did not considerably affect the oxygen consumption, membrane potential and NAD(P)H steady state. 

In the presence of CIII inhibitors and MB, the addition of ADP decreased the OCR. We may explain this phenomenon by the depolarizing effect of ADP. MB added before the ADP partially repolarized the mitochondria (Figure 2 and Figure 3). The entry of ADP via the ANT could depolarize the inner membrane, since the transporter is electrogenic [38]. Depolarization would decrease the driving force for attracting MB to enter the mitochondria and MB taking up electrons from either the matrix or the inner membrane. Decreased availability of MB for the reduction was well reflected in the increase in the NAD(P)H/NAD(P)^+^ ratio and the decrease in the OCR. The decreased OCR could be attributed to the phenomenon that less reduced MB was available to bypass the inhibition of CIII and reduce cyt c. The entry of ADP through ANT assumes an exchange with ATP, therefore ATP production is a necessary prerequisite for the function of ANT. There are two possible ways for ATP production in mitochondria with compromised OXPHOS machinery. One of them is the function of the adenylate kinase isoenzymes [39,40,41], which also operate in the matrix. The other route is the substrate-level phosphorylation (SLP) in the TCA cycle. SLP does not require efficient proton pumping or high membrane potentials, it only demands the oxidation of reducing equivalents to maintain an active TCA cycle. MB is able to support the provision of the oxidized coenzymes to further run this cycle.

## 5. Conclusions

**Figure 10:**
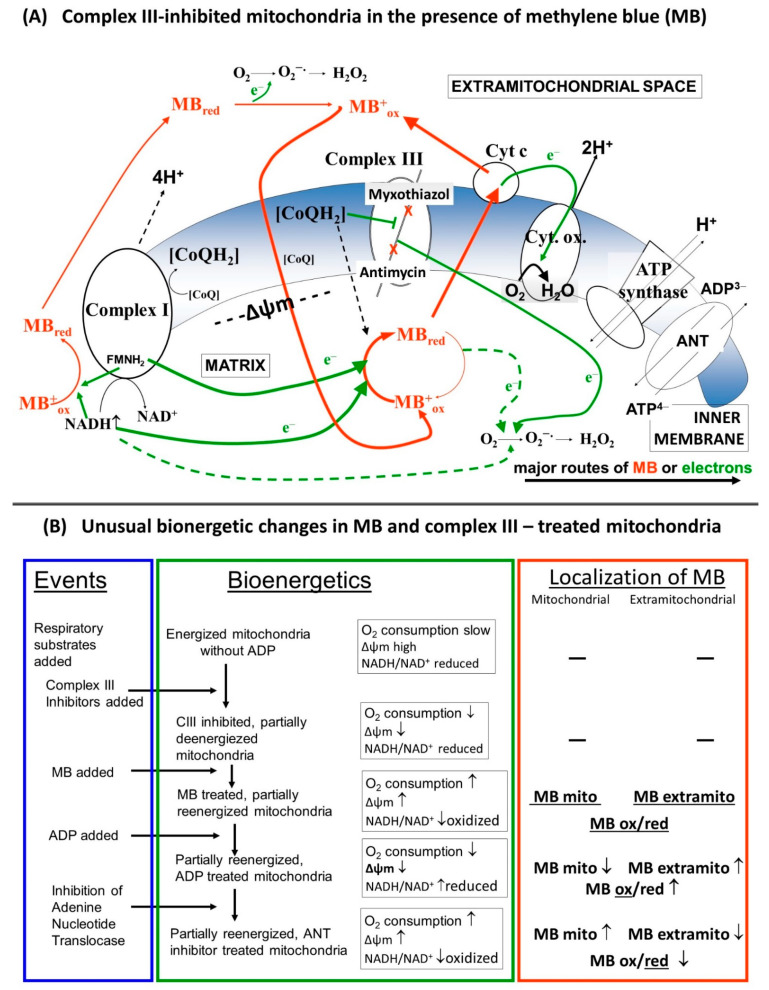
(**A**) Schematic representation of the putative charge-dependent redox-cycling mechanism of MB on the two sides of the inner mitochondrial membrane, (**B**) Detailed description of the alterations in the bioenergetic parameters.

This work is concerned with the bioenergetic effects of methylene blue (MB) in respiratory complex III-inhibited mitochondria. Evidence is provided in favor of the hypothesis that MB is indeed capable of transferring electrons from the respiratory chain and/or reducing equivalents directly to cyt. c, bypassing the complex III. Experiments were performed in three different rodent species in order to avoid individual species-specific characteristics. Unexpected bioenergetic phenomena are presented in terms of mitochondrial oxygen consumption, membrane potential and NADH steady state in the presence of MB and complex III inhibitors. These phenomena might be accounted for by the dynamic charge- and membrane potential-dependent changes of MB’s extra and intramitochondrial localization (Figure 10).

We have already entered the era of genome sequencing, where the molecular background of diseases associated with non-characteristic symptoms, with ill health, can be identified. Selected so-called rare diseases, like some mitochondrial diseases, are apparently not as rare as we earlier thought, and a relatively well tolerable drug like MB (or its selected analogues) is at times indeed capable of ameliorating the clinical pictures. On the other hand, unusual or rarely described bioenergetic features, like those described in this study, might stimulate thinking and dispute, and give rise to a better understanding of the true effects of selected redox compounds.

## Data Availability

Data can be found here: http://real.mtak.hu/120103/, accessed on 2 December 2020.

## Supplementary Materials

The following are available online at https://www.mdpi.com/2076-3921/10/2/305/s1.
