# Supplementary material for: Methylene Blue Bridges the Inhibition and Produces Unusual Respiratory Changes in Complex III-Inhibited Mitochondria. Studies on Rats, Mice and Guinea Pigs
Source: Antioxidants (Basel). 2021 Feb 16;10(2):305. doi: 10.3390/antiox10020305 (PMC7920423; doi:10.3390/antiox10020305)
Supplement: Supplementary file 1 [file antioxidants-10-00305-s001.pdf]

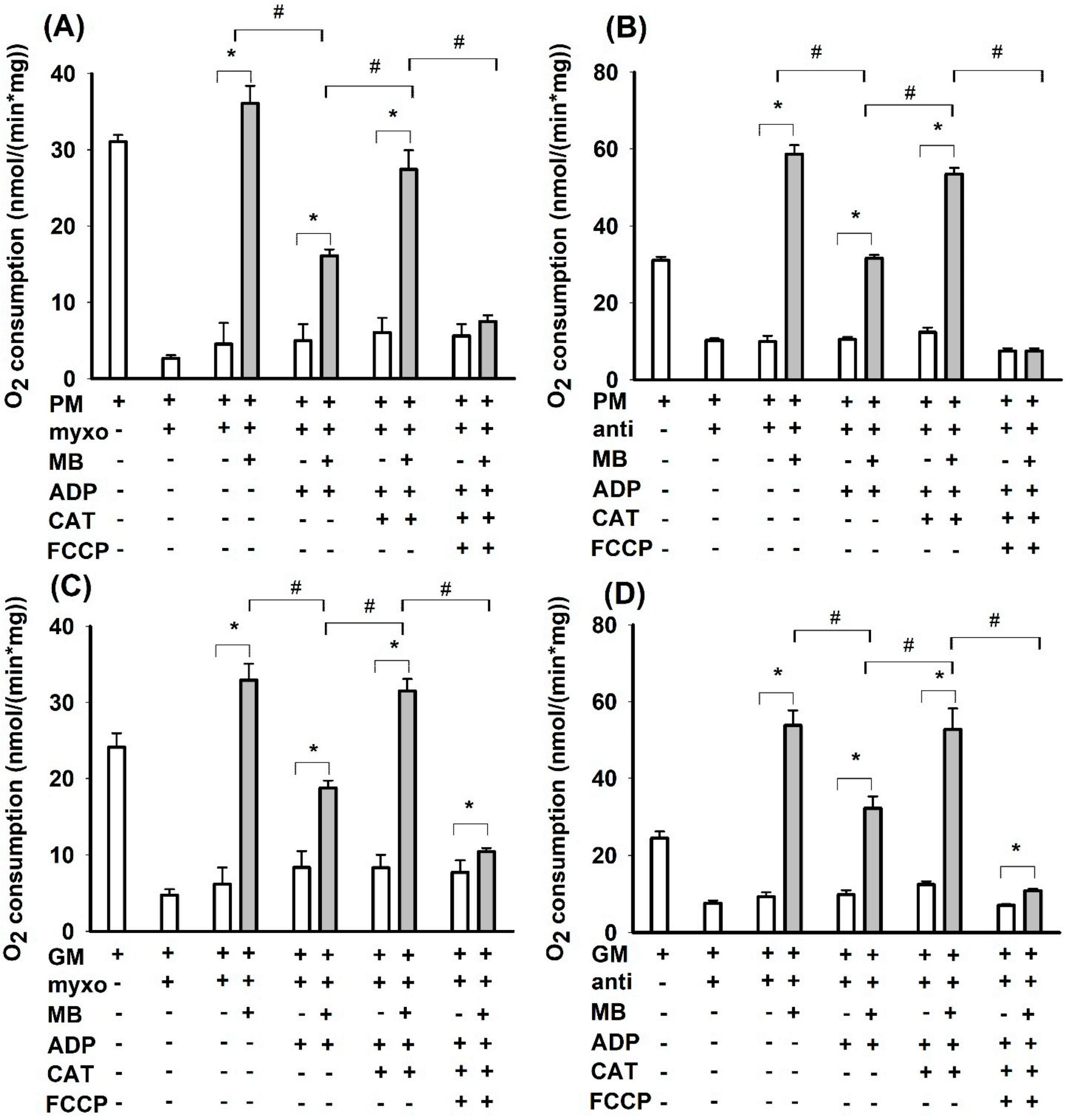

Figure S1. Effects of MB (2 µM) on the rate of oxygen consumption in complex III-inhibited guinea pig mitochondria;

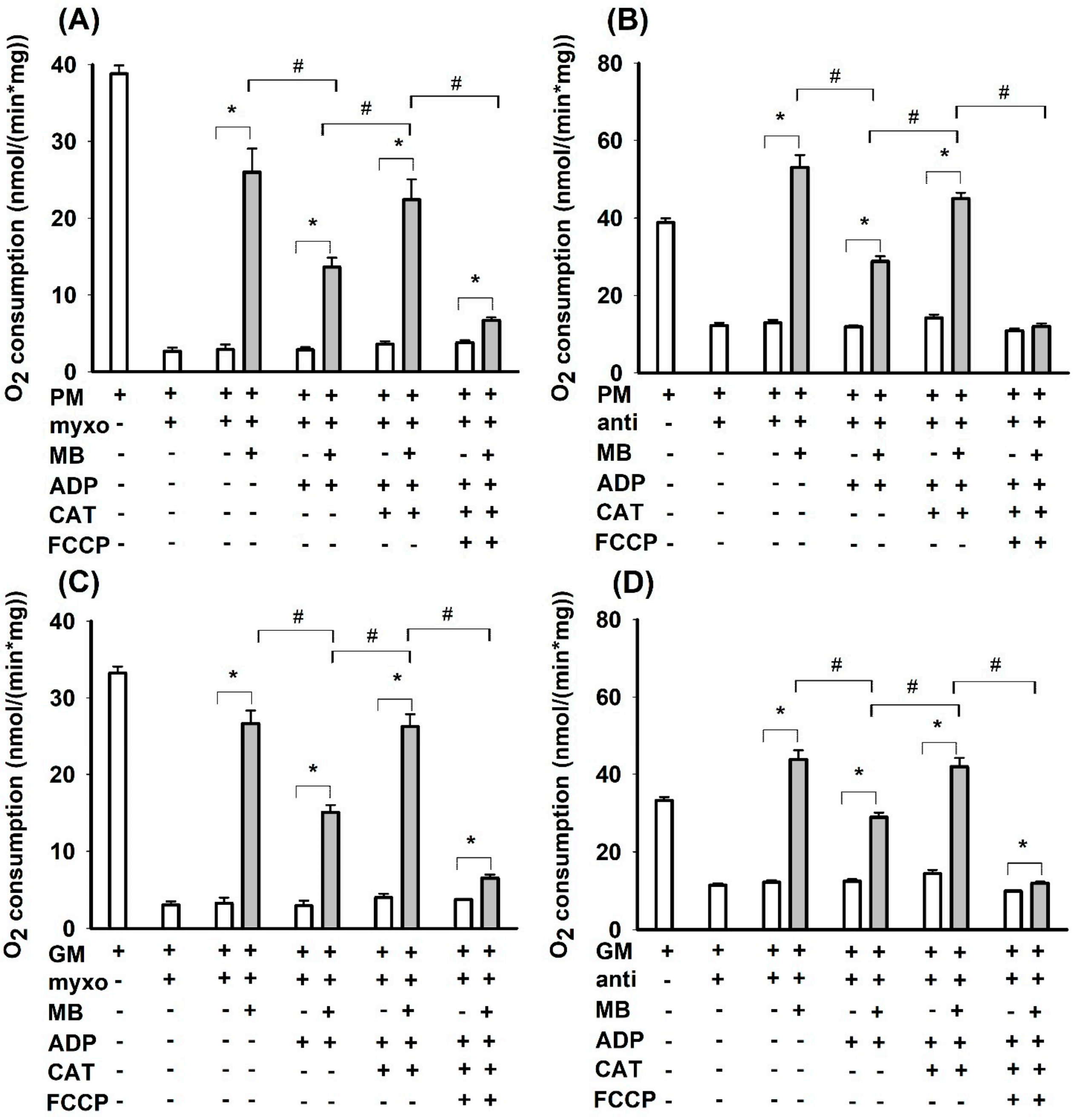

Figure S2. Effects of MB (2 μM) on the rate of oxygen consumption in complex III-inhibited rat mitochondria;

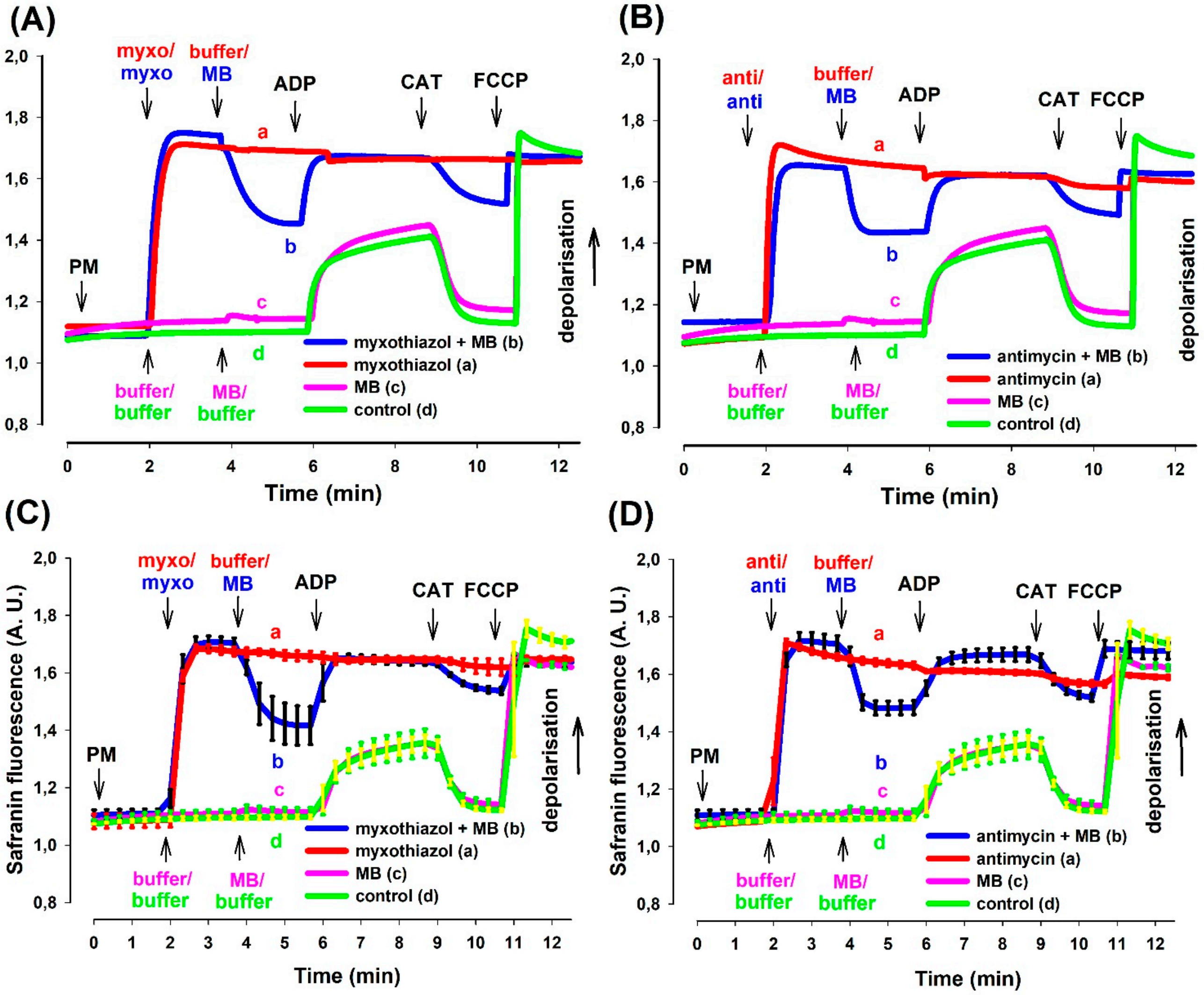

Figure S3. Effects of MB (2 μM) on the membrane potential in complex III-inhibited rat mitochondria;

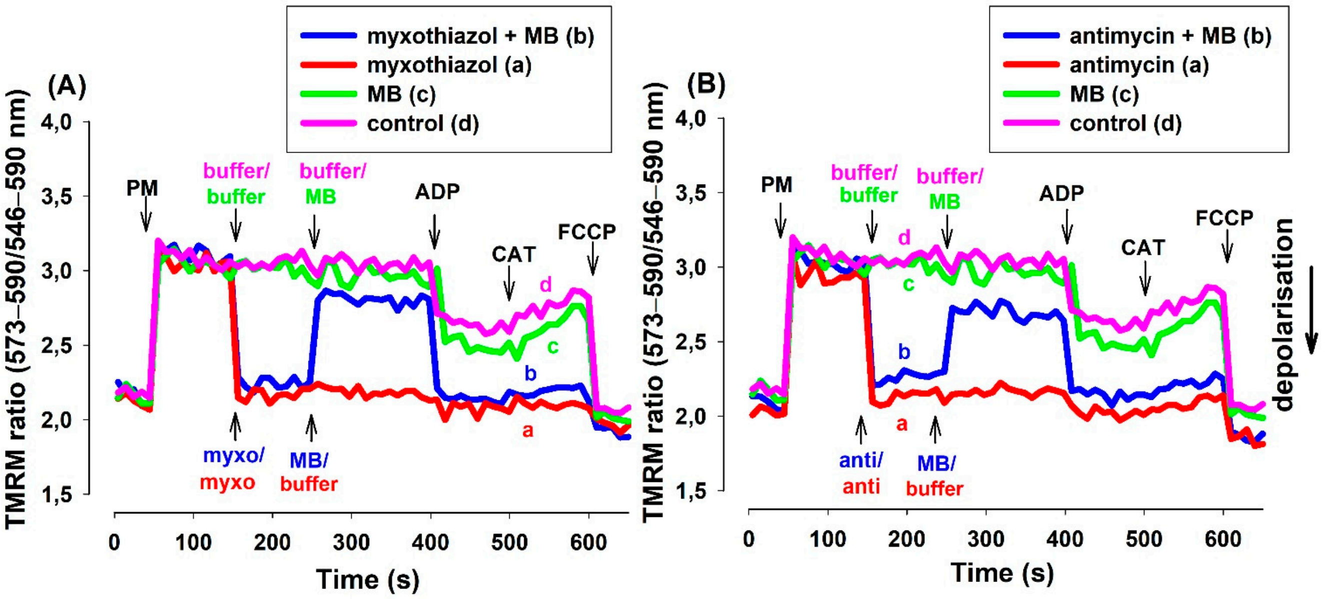

Figure S4. Effects of MB on the membrane potential (Δψm) of the complex III-inhibited (A: myxothiazol, B: antimycin) treated rat mitochondria;

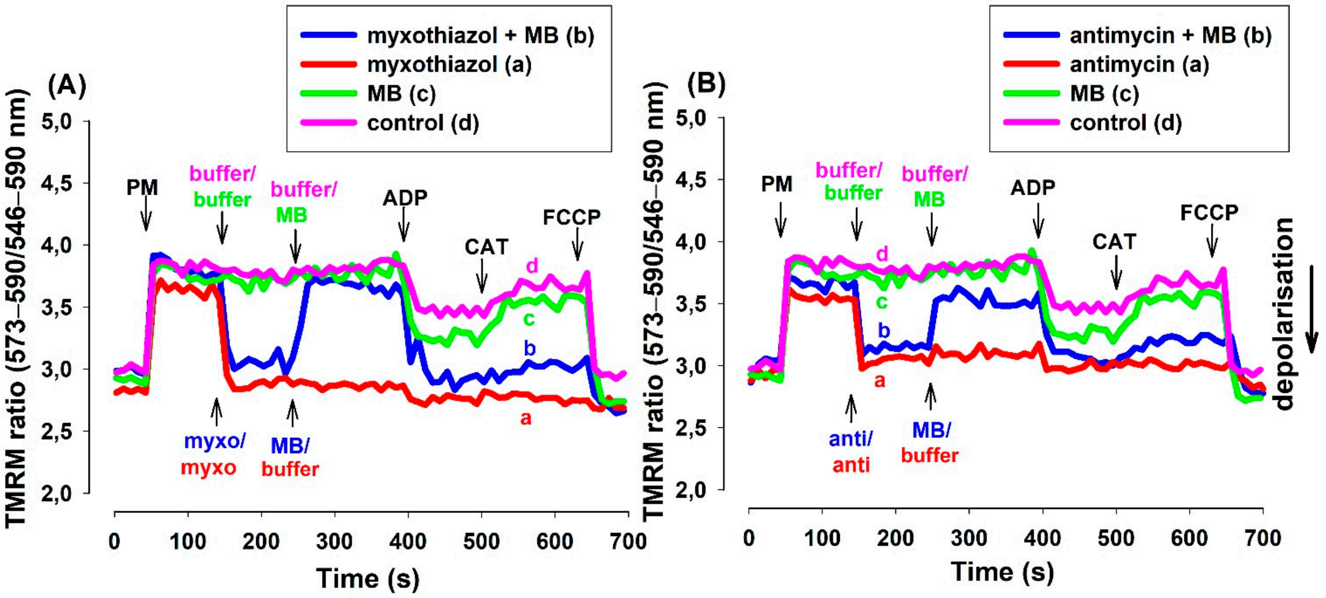

Figure S5. Effects of MB on the membrane potential (Δψm) of the complex III-inhibited (A: myxothiazol, B: antimycin) treated mouse mitochondria;

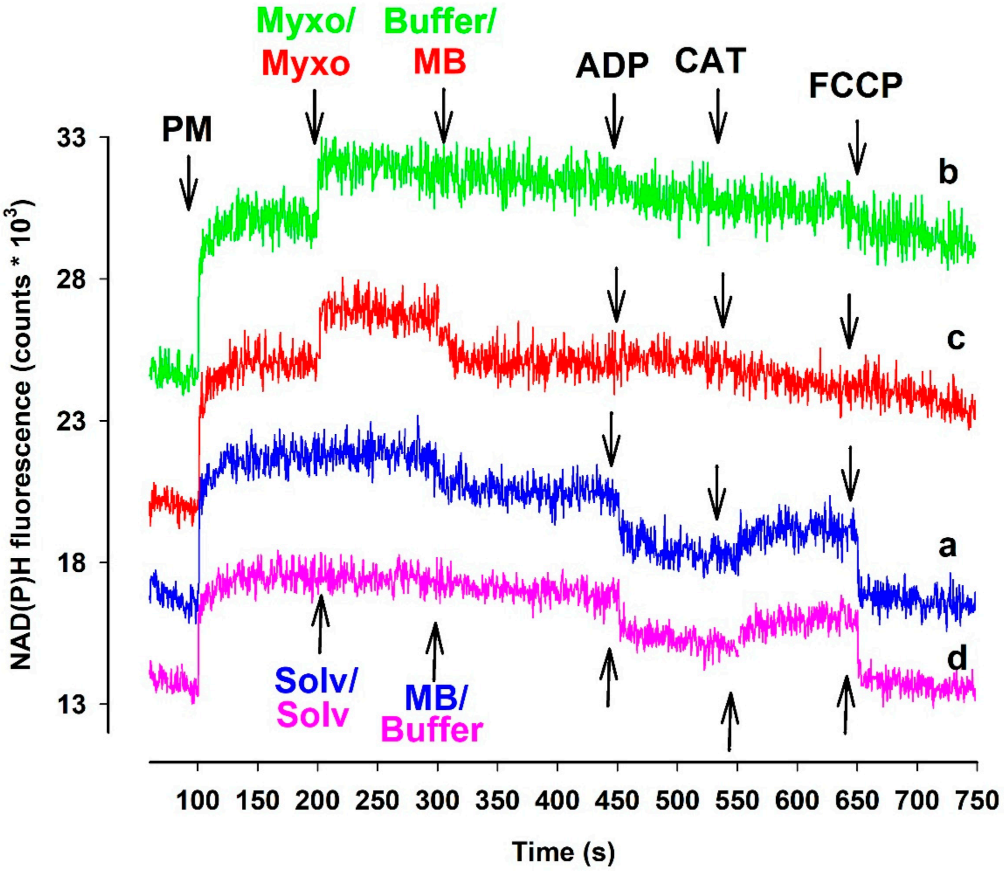

Figure S6. The effects of MB (2 μM) on NAD(P)H steady state of complex III-inhibited guinea pig mitochondria;

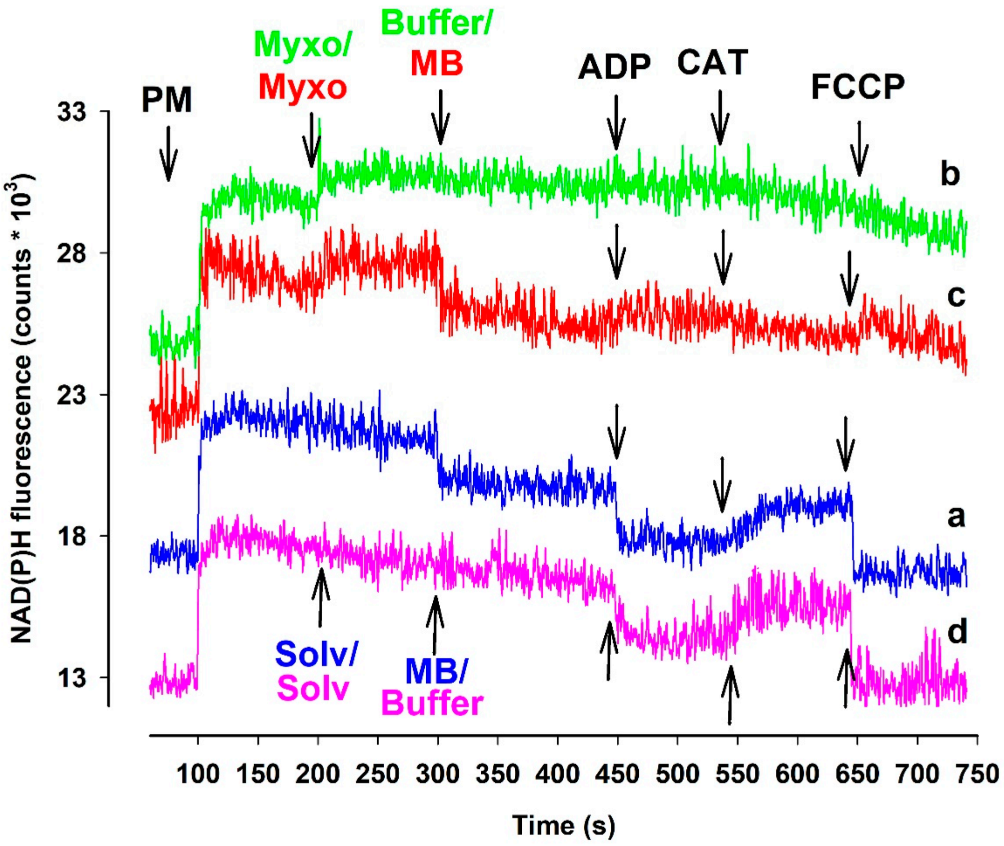

Figure S7. The effects of MB (2 μM) on NAD(P)H steady state of complex III-inhibited mouse mitochondria;

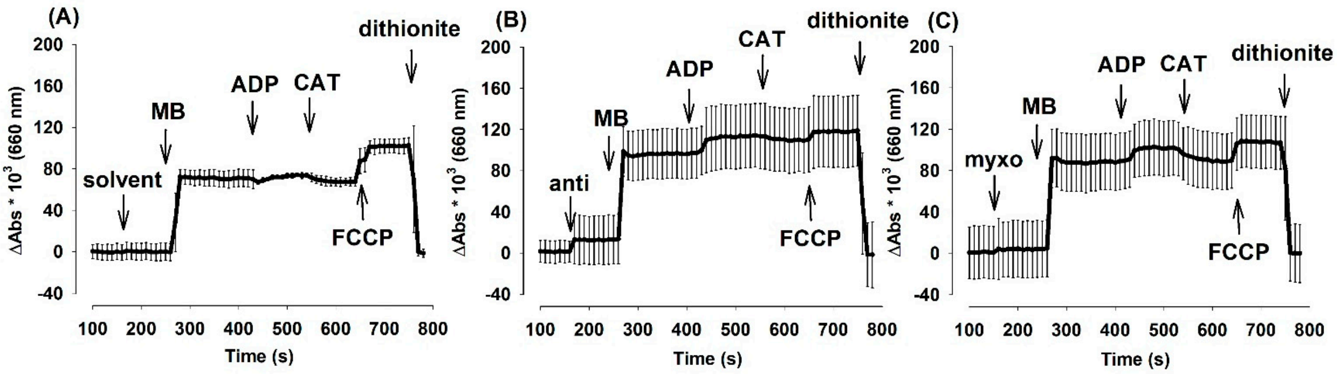

Figure S8. The effects of complex III-inhibitors on oxidoreduction steady state of MB (2 μM) in **guinea pig** mitochondria. Experiments were performed as described in Materials and Methods. In pyruvate *plus* malate-supported (5-5 mM) mitochondria absorbance differences (Δ Abs_{660} * 10^3) were detected in the presence or absence of MB in (**A**): uninhibited (**B**): myxothiazol (**C**): antimycin treated mitochondria. Further additions were as indicated. Curves represent the average of three independent experiments ± S.E.M.

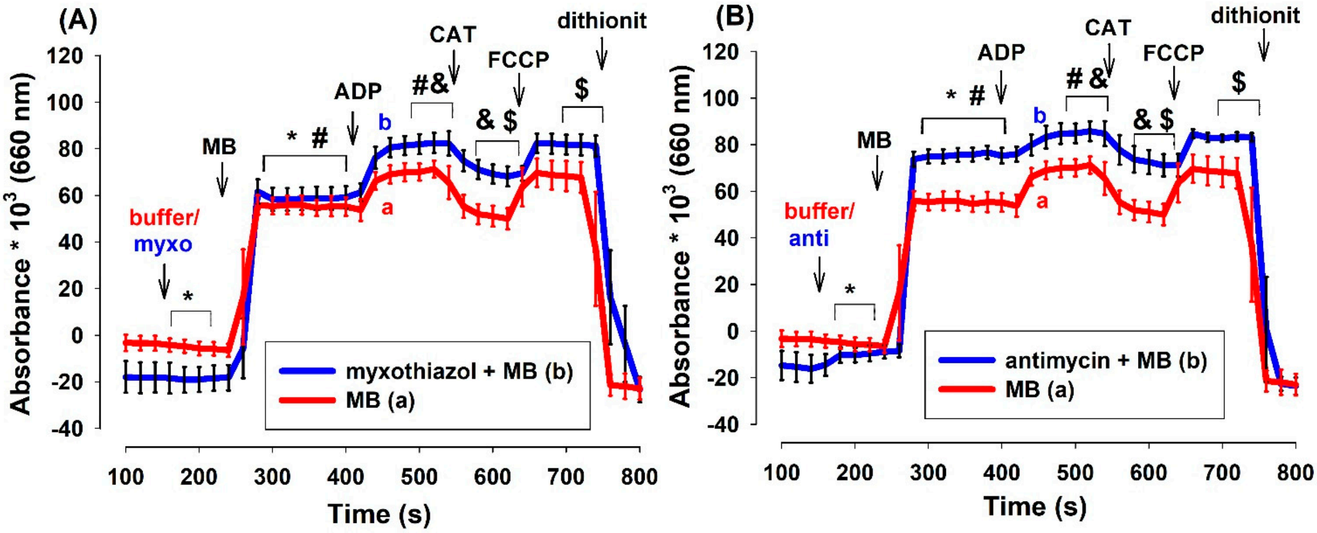

Figure 9. The effects of complex III-inhibitors on oxidoreduction steady state of MB (2 μM) in guinea pig mitochondria.
